# Feelings of Loneliness: Understanding the Risk of Suicidal Ideation in Adolescents with Internet Addiction. A Theoretical Model to Answer to a Systematic Literature Review, without Results

**DOI:** 10.3390/ijerph19042012

**Published:** 2022-02-11

**Authors:** Eugénie Khatcherian, Daniele Zullino, Diego De Leo, Sophia Achab

**Affiliations:** 1 Addiction Division, Department of Psychiatry, University Hospitals of Geneva,1202 Geneva, Switzerland; eugenie.khatcherian@hcuge.ch (E.K.); daniele.zullino@hcuge.ch (D.Z.); 2 Department of Psychiatry, Faculty of Medicine, University of Geneva, 1206 Geneva, Switzerland; 3 Department of Psychology, Primorska University, 6000 Koper, Slovenia; d.deleo@griffith.edu.au; 4 AISRAP, Griffith University, Mt Gravatt Campus, Mount Gravatt, QLD 4122, Australia; 5 De Leo Fund, 35137 Padua, Italy

**Keywords:** suicidal ideation, adolescent, internet addiction, loneliness

## Abstract

The Internet has become an essential tool for adolescents. It is part of their social integration within peers and supports their identity construction. The Internet can also become a source of addiction, especially when used as a coping strategy towards unpleasant life situations. Addiction to the Internet is often linked with an increase in the feeling of loneliness. The feeling of loneliness is an emotion present during adolescence. However, in excess, it can lead to suicidal ideation. Addiction to the Internet is also linked to an increased suicide risk. We questioned ourselves on the impact of the feeling of loneliness on the link between an excessive use of the Internet by adolescents and the risks of suicidal ideation. We attempted to find an answer to this question by performing a systematic review of the literature. We found no result matching our search criteria. We noted the absence of studies with regards to the interaction between the feeling of loneliness, addiction to the Internet and the risk of suicidal ideation amongst adolescents. We established a theoretical model which could be used as a lead for future research. We insist on the importance that studies need to be conducted in this domain, in order to enable us to establish efficient preventive measures for the risks of suicidal ideation amongst adolescents.

## 1. Introduction

### 1.1. Identity Construction at the Adolescence

Adolescence is marked by the passage from childhood to adulthood. Adolescents are confronted with physical transformation as well as the emergence of sexuality. They must appropriate their new body, face new impulses, all while developing their identity as adults in the making [1]. These processes can only happen by distancing themselves from the parenthood images, enabling adolescents to construct a different identity from their parents [1,2]. Adolescents weakened by their constructing self-reflection will seek landmarks and support from peers. Adolescents are marked by the need to belong to a group of peers offering identification models, which they rely on to build their identity, all while developing their singularity [1].

The adolescent transition is also characterized by a greater sense of loneliness [2,3].

### 1.2. Loneliness at the Adolescence

Loneliness is a subjective mental state of individuals with a significant deficit in their relationship to others [2,3]. The feeling of loneliness throws back to their own perception of their capabilities to create and/or maintain social ties [2]. This deficit can be quantitative or qualitative. It is relative, emerging from the comparison made by subjects towards the social norms, the ideal sociability they aspire to, as well as the one from their entourage [2,3]. 

It is crucial to differentiate loneliness and social isolation. Social isolation is an objective state and designs a quantifiable lack of social proximity and engagement with others. A socially isolated individual may not feel loneliness. Conversely, an individual with a lot of social interactions may have a feeling of loneliness [2,3].

Distancing from parental identity models during adolescence induces a feeling of loneliness. This confrontation with loneliness enables adolescents to gain autonomy, to think by themselves, make their own decisions and affirm themselves as individuals [2].

Adolescents are in a twofold movement. On one hand, they seek to isolate themselves from the adult world, while simultaneously seeking the company of their peers to fulfil their needs for attachment and belonging. Friendship will allow them to share emotions and the ongoing transformations upheavals. However, the quest for a group of peers to integrate with can be a tedious one. It requires finding peers with whom the adolescent will find similarities, share affinities and create a trust-building relationship. If the adolescent fails to find a group of peers, this will be accompanied by an exacerbated feeling of strangeness and loneliness [1,2].

We know adolescents with an avoidance or anxious attachment disorder possess fewer social skills and are more at risk of feelings of loneliness [4,5]. We can assume their deficit in social skill renders their quest for a group of peers even more complex.

Studies show that adolescents with a strong feeling of loneliness have a greater risk to develop suicidal ideation and/or behaviors [3]. The feeling of loneliness constitutes a predictor to the suicide risk [3,6,7].

### 1.3. Suicidal Ideation and Dying by Suicide in Adolescence

Suicide is an important public health issue, especially in adolescents. Suicide is the second cause of death worldwide for individuals aged between 15 to 29 years old [8]. As observed, adolescence is a period of mental fragility. The feeling of loneliness lived during adolescence can, if exacerbated (by the lack of a group of peers, for example), lead to suicidal ideation or even acts of suicide [2]. Loneliness is an important risk factor in the building up of suicidal ideation or in suicide attempts [3,7]. The Interpersonal Theory of Suicide by Thomas E. Joiner (2005) points specifically to the lack of feelings of belonging as one of the main risk factors in the construction of suicidal ideation [9]. In this theory, Thwarted Belongingness and Perceived Burdensomeness (the perception of being a burden on others) are the two factors that would lead to active suicidal ideation [9,10]. 

A study performed in 23 European countries went even further, by demonstrating that individuals who were living within communities based on mutual aid registered less suicides [11]. As members of these communities, individuals perceive themselves as being part of the group and therefore useful to others. Others rely on them, and they rely on others to solve problematic situations which may arise [11]. These elements converge with the observation in the literature of the reduction in the risk of suicide among adolescents who benefit from social support from their family and friends [12,13]. We can infer that the adolescent, well integrated in a family structure and a group of friends based on trust and mutual aid, will feel a sense of belongingness and a weak sense of burdensomeness.

### 1.4. Usage of the Internet by the Adolescent and Addiction to It

Adolescents are the part of the population with the highest prevalence of internet use. The internet offers a large diversity and variety of occupations and experiences. It has become an information, learning support, discovery, communication and entertainment tool [14].

For adolescents, the Internet constitutes space in which to extend their identity construction. As a matter of fact, it is a space where they can gather outside of parental oversight and shape to their own norms, with a common shared language and preferences [1]. Within this space, the adolescent may find the feeling of belongingness to a group and identification figures. The Internet will also offer the opportunity for the adolescent to create virtual identities: avatars which they shape towards the image they wish to show. This virtual construction is part of the process of identity construction of the adolescent, enabling them to experiment with whatever they wish to be and whoever they can be with their peers [1,15].

For adolescents in industrialized countries, the internet has become an essential tool in their identity development and socialization.

However, the use of the internet can also have negative consequences, particularly the development of an addiction. A meta-analysis from 2014 which reviewed studies from 31 nations reported that the global occurrence of internet addiction was estimated at 6% [16]. Adolescents are the part of the population with the highest risks of presenting an addiction to the internet [17,18]. The prevalence of internet addictions varies from one region to another. In Europe, the prevalence varies between 1.2% and 11.8% [19]. In China, the prevalence varies between 9.56% and 24% [19]. We found a 20.6% prevalence in the USA, and 18% in Canada [19]. In South Korea, it reaches 30% of adolescents being at risk of Internet and smartphone addictions and in Japan, 23.7% of adolescents are classified as internet addicts [19]. The vast differences in prevalence rates between jurisdictions are explained by the difference in the criteria used in the diagnosis, as well as the difference in age and culture of the studied group of the population [18]. 

Despite the absence of consensus, the literature provides a definition of the addiction to the internet as being an excessive usage characterized by a lasting and significant impairment on at least one aspect of the life of the individual (social, familial, academic, professional) [20]. 

Wrongfully, we could have a tendency to consider the time spent online as a diagnosis element. However, for behavioral addictions, this does not enable the differentiation between a committed use from an addictive use [21]. The COVID-19 pandemic lockdown periods are extremely representative of this. Adolescents had to spend more time online for their scholarship, as well as to keep in contact with their friends, without this becoming an issue as it was a practical response to the situation. Therefore, the loss of control on a behavior, even in the face of dire consequences on the functioning of the individual, appears to be a more relevant element. 

The concept of internet addiction covers a very heterogeneous set of problematic behaviors, as a number of devices can be used, (such as computers, smartphone, tablets, gaming consoles, etc.) with extremely variable and fluctuating usage (social media, video games, sexual content, video viewing, information retrieval…) [18]. The internet is the vector of potential addictive patterns of use of some online activities such as gaming and gambling; these two disorders have recently been recognized by WHO in ICD-11 draft as addictive behaviors [22].

Despite these vast variations in the prevalence and behaviors, addiction to the internet is a real public health issue, as concluded in the WHO meeting of August 2014 on the topic of the impact on public health of excessive use of internet, computers, smartphones and similar electronic devices [18]. This meeting performed a review of available evidence and established the main direction for future research to be made. Experts have concluded that given the extent of the negative repercussions on health, and the high rates of comorbid psychiatric disorders including suicidal behaviors, effective prevention policies are needed [18].

Internet addiction is often associated with comorbid psychosocial issues (i.e., self-isolating, feeling of loneliness, depressive mood, suicidal behaviors, anxiety disorders, poor academic performance) and other health issues (i.e., vision, sleep, sedentary lifestyle, musculoskeletal disorders) [18,23,24].

Multiple elements that are able to lead an adolescent towards an internet addition have already been identified in the literature.

The first one is going online to compensate for a negative life situation and associated emotions. The internet can then play one or more of several roles [15,25]:

- It can be a means to escape from reality, to flee negative situations and emotions. This behavior is described in the literature as escapism;
- It can be a means to find online what the adolescent cannot find in real life;
- It can be a means to replay in an online space difficulties encountered in real life, in order to find means to overcome them;
- It can be a place of sublimation for negative emotions.

The second element is the usage of the internet by adolescents with a greater impulsive drive, in a constant quest for novelty and short-term reward [26,27,28]. Within that group of adolescents, most notably found are those suffering from ADHD [29].

The literature highlights links between adolescents suffering from loneliness, depression and anxiety with their use of the internet as a coping strategy [24,29,30]. Adolescents suffering from relational difficulties make use of the internet to compensate for their deficits in social capital, to fill a lack of social support and are more prone to develop internet addiction [13,30,31]. The internet offers less risky possibilities for social interactions than face to face interactions, due, in large part, to the feeling of anonymity it provides [32].

However, the literature points out that addiction to the internet tends to increase social isolation and the feeling of loneliness [18,23,32]. Indeed, individuals will favor online activities rather than offline activities which could enable them to create social ties [32,33]. Kraul et al. name this effect the displacement theory [33].

The other element pointed out in the literature is the impact of addictive usage of the internet on the other fields of social integration of individuals: absences, family conflicts, lack of involvement in work, school, other social activities, etc., which will, in the long term, favor social isolation and the feeling of loneliness [32].

Two studies highlight the possibility of increased feelings of loneliness and increased suicidal thoughts for adolescents with either low or excessive usage of internet or social media as opposed to those with a moderate usage [24,34]. These elements highlight the existence of an appropriate investment range for the internet and social media, as well as the importance these tools have taken in the lives of adolescents. These tools are necessary for their good socialization in the group of peers. With regards to addiction to the internet, we deduced that the more adolescents invest excessively in the internet, the more they will present risk of feeling loneliness as well as having suicidal ideation.

### 1.5. Research Question

Adolescents suffering from a feeling of loneliness have an increased risk of presenting an addiction to the internet due to their usage of it as a coping strategy [24]. Addiction to the internet is linked to an increased risk of suicidal ideation or behavior [24]. Furthermore, loneliness is also a predicting element, for the suicide risk and the addiction to the internet frequently causes an increase in the feeling of loneliness [3,23].

However, not all adolescents suffering from an addiction to the internet have suicidal ideation. We are asking ourselves to what extent is the feeling of loneliness linked to the adolescents’ addictive use of the internet and the risk of suicidal ideation.

The answer to this question would allow a better understanding and prevention of the suicide risk of adolescents suffering from an internet addiction. It is crucial to question the repercussions of the COVID-19 pandemic and its health restrictions on the mental health of adolescents. Indeed, the adolescent population suffered from an increase in the feeling of loneliness that naturally led to an increase in the usage of connected tools as a means to maintain social bounds and as a coping strategy towards negative emotions [35,36,37]. Simultaneously, studies show an increase in the number of suicides and suicidal ideation amongst adolescents [36].

## 2. Materials and Methods

To answer this question, we performed a systematic review of the literature. A comprehensive literature review was conducted using the following three online databases:

- Scopus;
- Pubmed;
- PsycINFO.

The searches were performed on 20 October 2021 with the following keywords on Pubmed and PsycINFO: (adolesc* OR teen* OR pubesc* OR youth*) AND lonel* AND suicid* AND (“problematic use of internet” OR “excessive use of internet” OR “internet addiction”) and the following keywords on Scopus, as Scopus does not allow an asterisk: (adolescence OR adolescent OR teen) AND loneliness AND (“suicidal ideation” OR suicide) AND (“problematic use of internet” OR “excessive use of internet” OR “internet addiction”).

On Scopus, we limited our search to literature reviews and research articles. On Psycinfo we limited our search to Journal article.

The result of our searches on the different online databases were:

- 92 hits on Scopus;
- 3 hit on Pubmed;
- 0 hit on PsycInfo.

**Figure 1:**
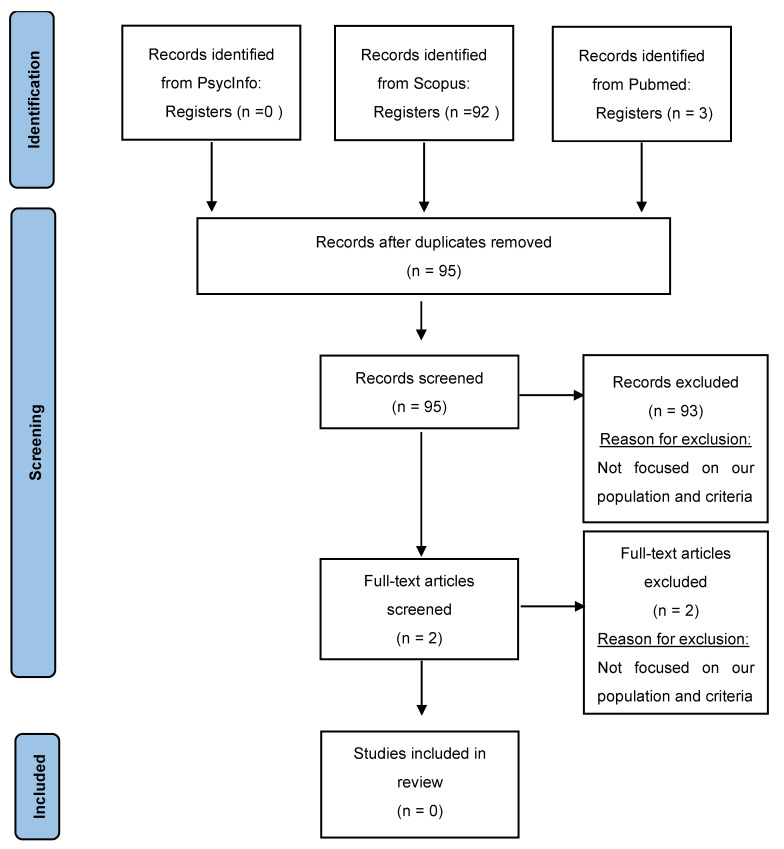
PRISMA’s flow diagram of study selection processes.

There were no duplicates identified in the results. We performed a preselection based on the titles and abstracts of the search results, with the following inclusion criteria: (1) we targeted the adolescent population with internet addiction (including the various addictive online behaviors: gaming, gambling, social networking, cybersex, etc.); (2) which approached the implication of the feeling of loneliness in the development of suicidal ideation; (3) we did not put restrictions on the publication date or the location of the observed population; (4) published in English or French. Studies were excluded based on the following exclusion criteria: (I) assessing behaviors other than internet addiction; (II) did not focus on the adolescent population; (III) deriving from sources other than peer-reviewed journals (e.g., non-peer-reviewed journals, conference abstracts, chapters, books). In total, 28 studies were excluded because they did not focus on the adolescent population; 36 studies were excluded because they did not focus on internet addiction; 29 studies were excluded because they did not focus on the relationship between the feeling of loneliness and the development of suicidal ideation. Two articles were found to be relevant. The final selection was made through a complete review of each article with the same inclusion and exclusion criteria. None were retained. One article did not focus on the feeling of loneliness, but on interpersonal problems, and the other one did not target internet addiction but internet use in general [38,39]. The Figure 1 shows the study selection processes following the flow diagram of the Reporting Items for Systematic Reviews and Meta-analyses (PRISMA) 2020 Statement [40].

Given the absence of results, we decided to perform another search on the ongoing research on this topic. We performed on 27 October 2021, a search in the clinical trial register using the following keywords: (adolesc* OR teen* OR pubesc* OR youth*) AND lonel* AND suicid* AND (“problematic use of internet” OR “excessive use of internet” OR “internet addiction”). The search did not return any result.

## 3. Results

There were no results found in the literature. The article “Relationship between adolescent suicidality, self-injury and media habits” from Erin L. Belfort and Lindsay Miller was excluded as it targets the media habits of adolescents without specifically addressing adolescents’ addictive use of the internet [38].

However, certain elements of this article are interesting to point out. Belfort and Miller mention the hypothesis of some researchers that cast aside or socially anxious adolescents would benefit from online platforms to create their social network. However, they also mention findings which reveal that adolescents with communication skills would increase the social capital and enrich their social life via social networks, while the ones with fewer communication skills would not be able to increase their social capital. Furthermore, the latter would be confronted by more pervasive feelings of loneliness [38]. 

The article underlines that adolescents which belongs to a social minority, defined by a peculiarity distinguishing them (such as the sexual orientation, a mental illness, etc.) would be able to find communities with whom to share their experiences and difficulties, without being stigmatized. Therefore, in these situations, social networks enable the usually excluded adolescents in enriching their social ties [38].

The article mentions the importance of the type of platform used. The content to which the adolescent will be exposed, as well as the specific impact it can have on a given individual. Adolescents can use social networks in an active or passive manner.

In active usage, the adolescent is actively interacting with others on social networks, whereas in the passive usage, the adolescent is simply scrutinizing the social networks without interacting with other people. Active usage is often linked with a decrease in feelings of loneliness, while passive use generally produces an increase in feelings of loneliness. [38].

Belfort and Miller’s article also mentions the contagion phenomenon or incitation to suicidal behaviors on the Internet. Adolescents are extremely sensitive to the contagion phenomenon. The literature noted that the exposition to suicidal behaviors amongst the inner circle of the adolescent or via the media increased the risks of suicidal behavior for adolescents. Moreover, Belfort and Miller specify that one study differentiated the exposure to the announcement of a suicide on a social media or on an online forum. The exposition to the announcement of a suicide via social media is not linked to an increase in suicidal ideation, whereas the exposition of such an announcement on an online forum is. Belfort and Miller mention the hypothesis from the literature that the degree of “connectedness”, or at the very least, the “perceived connectedness” is a crucial element to the comprehension of the impact of the information to which the adolescent is exposed [38]. The other article which we found to be interesting was: The relationship between mobile phone use and suicide-related behaviors among adolescents: the mediating role of depression and interpersonal problems. This study highlights how a high-intensity mobile phone use is indirectly associated with the risk of suicide-related behaviors through interpersonal problems. Unfortunately, this study did not measure the feeling of loneliness perceived by participants [39].

## 4. Discussion

### 4.1. Insufficient Literature

The absence of articles dealing with the links between feelings of loneliness, addictive usage of the Internet and suicidal ideation was somehow surprising. Several reasons can explain this absence in the literature: our first hypothesis is that addiction to the Internet is a recent concept, which is still controversial amongst the scientific community [18]. Furthermore, we noticed the concept is still unrecognized and vague for a vast number of healthcare professionals. The distinction between a passionate or pathological behavior is still under discussion by experts [38].

We also form the hypothesis that as the addiction to the Internet covers a vast variety of behaviors, it is all the more complex to study the complete set of these behaviors as well as forming a global conclusion. 

### 4.2. Knowledge from the Literature

We propose to start from the knowledge identified in the literature and observed in our everyday practices to make a theoretical hypothesis, which can be used as a basis for future research. 

#### 4.2.1. Internet Investment by Adolescents with Feelings of Loneliness

We have previously seen that adolescence is a critical period in which adolescents are in their identity construction phase and in need to find a group of peers; friends which they can identify themselves with and by which they feel recognized as individuals [1].

Adolescents with fewer social skills will have difficulties in building solid relationships and finding a group of peers, and this will increase their feelings of loneliness [4,5]. As a consequence, they will seek social ties (more) actively on the Internet, to compensate for their deficiency in real life social capital [38].

#### 4.2.2. The Adolescent’s Possibility to Create Social Ties via the Internet

The usage of the Internet appears to be a determining element. For the adolescent, it represents the possibility of creating and strengthening social ties with peers [38].

Different elements are identified in the literature as influencing the capabilities of an individual in creating social ties online:

- The kind of social usage made by the adolescent of the Internet, socially active or socially passive, can lead to different outcomes [38]. A socially active usage—rich in communications and interactions with peers—could decrease feelings of loneliness. On the contrary, a socially passive usage—in which one scrutinizes the social network activities of their peers without interacting with them, or playing lonely without interacting with others—could increase the feeling of loneliness [34,38,41].
- The kind of social usage, active or passive, will depend on the social skills of the individual. The literature finds that adolescents with communication skills would increase their social capital and enrich their social life via social networks, while the ones with little communication capabilities would not be able to increase their social capital and would increase their feelings of loneliness [38]. An article highlights the same outcome regarding the use of video games. It explains that adolescents with a secure attachment will be those with the most socially beneficial video game behavior and therefore benefit the most of an increase in social satisfaction, perceived peer support, sense of self-worth and belonging [42].
- Additionally, we question ourselves on the impact of the specific social platform used, as it can favor or be detrimental to more socially active usage and therefore the feeling of belonging to a group [38]. We can also infer that adolescents choose specific platforms according to their attachment style, and therefore their abilities to create social ties.

### 4.3. Hypothesis for a Theoretical Model

We have observed that adolescents with fewer social skills have more difficulties creating social ties and then finding a group of peers. These difficulties make them more prone to experiencing the feeling of loneliness [4,5]. The feeling of loneliness is an important risk factor in the development of suicidal ideation. 

We have also seen that adolescents suffering from the feelings of loneliness will have higher risks of presenting an addiction to the Internet, as they are using the Internet as a coping strategy [13,30,31,38]. Indeed, the Internet can represent a space in which to find sufficiently satisfying social ties, even more so when adolescents are unable to find these in real life. Furthermore, adolescents suffering from a lack of social skills will feel safer in online relations as the Internet offers a sense of anonymity. The Internet can also constitute an escapist coping strategy towards negative emotions such as loneliness [13,30,31,38].

We hypothesize that if the use of Internet allows adolescents to create sufficiently satisfying ties with their peers, it will lead to a decrease in their feelings of loneliness, as well as the risk of having suicidal ideation. Sufficiently satisfying social ties allow the adolescent to feel a sense of belonging to a group of peers, a group in which they can identify themselves, in which they can develop trust, with whom they can share their experiences and difficulties while feeling supported and offer their support to others. We build our hypothesis from the elements seen previously, which show how individuals who are socially active online will feel a benefit in terms of a reduction in the feeling of loneliness, as well as an increase in social satisfaction, perceived peer support and the sense of self-worth and belonging [38,42]. This reduction in the feeling of loneliness will be a factor in the decrease in the risk of suicide or suicidal ideation.

As we saw previously, the possibility to create sufficiently satisfying social ties will depend on the structure of the platform used by the adolescent and the kind of social usage made by the adolescent (active or passive) [38,42]. The preference for certain platforms and their kind of social usage will be dependent on the social skills of the adolescent. 

Thus, the adolescents which turn to the Internet as a coping strategy to soothe their feeling of loneliness, which is secondary to their social skill deficit, will encounter more difficulties than others in creating social ties online [38,42]. 

If adolescents fail to find sufficiently satisfying social ties to fill their need for social exchange and support, including the feeling of belonging to a group of peers, they will persist in their loneliness. This new failure will intensify the adolescent feelings of social exclusion, perceived burdensomeness and self-depreciation, which can lead to stronger feelings of loneliness, boredom and eventually suicidal ideation. We have also mentioned in the introduction that addiction to the Internet could cause a deficiency in face-to-face relations, leading to social isolation with an increased feeling of loneliness due to the negative consequences on the other social spheres of the individual [32]. These adolescents will therefore experience an increase in their social isolation, as well as an increase in their feeling of loneliness [32]. Should these adolescents persist in an escapist coping strategy, favoring an addiction to the Internet while being unable to create social ties online, they will get caught in a vicious circle, increasing their feeling of loneliness and the risk of developing suicidal ideation. This concurs with the observation made in the literature between the degree of addiction to the Internet and the feeling of loneliness, which is a linear association [43].

**Figure 2:**
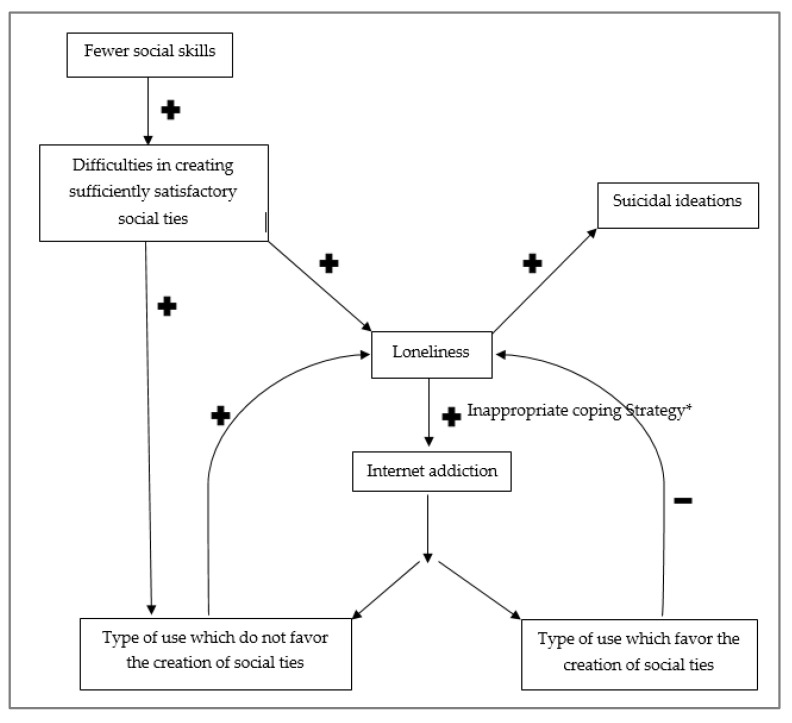
Theoretical model. The + represent a favored occurrence by the previous element and the—represent a disadvantaged occurrence by the previous element. * Inappropriate coping strategy could be escaping from adverse life-events or managing negative emotions.

Adolescents suffering loneliness could have the reaction to intensify their inappropriate use of the Internet as a coping strategy of escapism to avoid negative emotions. This behavior could lead to a vicious circle, increasing their feeling of loneliness further due to an inappropriate coping strategy [32] (see Figure 2).

This hypothesis implies that for good prevention of suicide risk in adolescents suffering from an addiction to the Internet, we must evaluate their feeling of loneliness and their capability to find a group of peers while taking into account their more or less socially active usage of the Internet. The prevention would not focus primarily on reducing the usage of connected tools, but rather working on the lack of social skills and the implementation of more efficient coping strategies towards the feeling of loneliness. 

These elements need to be considered in the current context of the COVID-19 pandemic and the impacts of health measures on the adolescent population. Many adolescents experienced feelings of loneliness and had to resort to the use of the Internet as a coping strategy. We can make the hypothesis that adolescents with the most difficulties in creating or maintaining social ties online have lived an absence in the soothing of their feeling of loneliness and therefore were more at risk of having suicidal ideation as well as escapism coping strategies in their use of connected tools. 

### 4.4. Limitations

Regrettably, the literature on the implication of the feelings of loneliness with the interaction between the addictive use of the Internet by adolescents and the suicide risk is very limited. Our literature review did not provide any results. In this paper, we have tried to build a theoretical model to understand the complexity of this interaction. Further research is needed to verify the validity of this model. We are aware that we have possibly identified only part of the complexity that may exist in the interaction between addictive use of Internet, feelings of loneliness typical of adolescence and the onset of suicidal ideation.

## 5. Conclusions

The Internet has become an integral part of our daily life, transforming the world of our relationships. Adolescents are the part of the population that is most exposed to these changes, which directly impact the entire process of adolescence.

Understanding the transformations that the Internet is bringing to the lives of young individuals is of crucial importance. Adolescent suicide prevention should pay particular attention to the impact of Internet addiction, with a view of integrating the impact of loneliness feelings. As highlighted throughout this article, feelings of loneliness are one of the key elements leading to the development of suicidal ideation; they are very frequent during adolescence.

Satisfactory social ties might enable adolescents to decrease their feelings of loneliness, therefore decreasing the risk of suicidal ideation. On the contrary, should they fail to create good-quality social ties, adolescents would be confronted by feelings of social exclusion and incapability; this may lead to an increase in feelings of loneliness and eventually to the development of suicidal ideation.

Therefore, capabilities of adolescents to create satisfactory social ties are crucial elements to take into consideration in suicide risk prevention.

Further research, aimed to better identify the processes in place in the interaction between feelings of loneliness, Internet addiction and suicidal ideation, could enable us to elaborate better suicide prevention solutions for adolescents, possibly by using their own preferred tool: The Internet.

## Data Availability

Not applicable.

## References

[1] Khatcherian E., Zdanowicz N. (2018). Why do cyberbullied adolescents stay in contact with their harasser? A literature review and reflection on cyberbullied adolescents’ Coping Strategies. Psychiatr. Danub..

[2] Dupont S. (2016). L’adolescent et l’épreuve de la solitude. Rev. L’enfance L’adolescence.

[3] McClelland H., Evans J.J., Nowland R., Ferguson E., O’Connor R.C. (2020). Loneliness as a predictor of suicidal ideation and behaviour: A systematic review and meta-analysis of prospective studies. J. Affect. Disord..

[4] Moeller R.W., Seehuus M. (2019). Loneliness as a mediator for college students‘ social skills and experience of depression and anxiety. J. Adolesc..

[5] Solmi M., Veronese N., Galvano D., Favaro A., Ostinelli E.G., Noventa V., Favaretto E., Tudor F., Finessi M., Il Shin J. (2020). Factors associated with loneliness: An umbrella review of observational studies. J. Affect. Disord..

[6] DiTommaso E., Brannen-McNulty C., Ross L., Burgess M. (2003). Attachment styles, social skills and loneliness in young adults. Personnal. Individ. Differ..

[7] Stickley A., Koyanagi A. (2016). Loneliness, common mental disorders and suicidal behavior: Findings from a general population survey. J. Affect. Disord..

[8] World Health Organization, Mental Health and Substance Use Team (2019). Suicide in the World. Global Health Estimates. https://www.who.int/publications/i/item/suicide-in-the-world.

[9] Joiner T.E. (2007). Why People Die by Suicide.

[10] Van Orden K.A., Witte T.K., Cukrowicz K.C., Selby E.A., Joiner T.E. (2010). The interpersonal theory of suicide. Psychol. Rev..

[11] Zadravec Šedivy N., Podlogar T., Kerr D.C.R., De Leo D. (2017). Community social support as a protective factor against suicide: Agender specific ecological study of 73 regions of 23 European countries. Health Place.

[12] Banstola R.S., Ogino T., Inoue S. (2020). Self-esteem, perceived social support, social capital, and risk-behavior among urban high school adolescents in Nepal. SSM-Popul. Health.

[13] Miller A.B., Esposito-Smythers C., Leichtweis R.N. (2015). Rôle of social support in adolescent suicidal ideation and suicide attempts. J. Adolesc. Health.

[14] Bremer J. (2005). The internet and children: Advantages and disadvantages. Child Adolesc. Psychiatr. Clin. N. Am..

[15] Achab S. (2019). Réflexions sur les enjeux psychiques du «always-on» chez les jeunes. Grandir à l’ère du Numérique.

[16] Cheng C., Yee-lam Li A. (2014). Internet addiction prevalence and quality of (real) life: A meta-analysis of 31 nations across seven world regions. Cyberpsychol. Behav. Soc. Netw..

[17] Chung T.W.H., Sum S.M.Y., Chan M.W.L. (2019). Adolescent internet addiction in Hong Kong: Prevalence, psychosocial correlates, and prevention. J. Adolesc. Health.

[18] World Health Organization (2014). Public Health Implications of Excessive Use of the Internet, Computers, Smartphones and Similar Electronic Devices Meeting Report. https://apps.who.int/iris/bitstream/handle/10665/184264/9789241509367_eng.pdf.

[19] Chi X., Hong X., Chen X. (2020). Profiles and sociodemographic correlates of internet addiction in early adolescents in southern China. Addict. Behav..

[20] Sela Y., Lev Bar-Or R., Kor A., Lev-Ran S. (2021). The internet addiction test: Psychometric properties, socio-demographic risk factors and addictive co-morbidities in a large adult sample. Addict. Behav..

[21] Brody A., Billieux J. (2020). Présentation. Sci. du Jeu.

[22] World Health Organization (2019). Betadraft of ICD-11. https://icd.who.int/browse11/l-m/en.

[23] Yao M.Z., Zhong Z.J. (2014). Loneliness, social contacts and internet addiction: A cross-lagged panel study. Comput. Hum. Behav..

[24] Lee S.Y., Park E.-C., Han K.-T., Kim S.J., Chun S.-Y., Park S. (2016). The association of level of internet use with suicidal ideation and suicide attempts in south Korean adolescents: A focus on family structure and household economic status. Can. J. Psychiatry.

[25] Achab S., Simon O., Müller S., Thorens G., Martinotti G., Zullino D., Khazaal Y., El-Guebaly N., Carrà G., Galanter M. (2015). Internet Addiction. Textbook of Addiction Treatment: International Perspectives.

[26] Mihajlov M., Vejmelka L. (2017). Internet addiction: A review of the first twenty years. Psychiatr. Danub..

[27] Jeong B., Lee J.Y., Kim B.M., Park E., Kwon J.-G., Kim D.-J., Lee Y., Choi J.-S., Lee D. (2020). Associations of personality and clinical characteristics with excessive internet and smartphone use in adolescents: A structural equation modelling approach. Addict. Behav..

[28] Dalbudak E., Evren C., Topcu M., Aldemir S., Coskun K.S., Bozkurt M., Evren B., Canbal M. (2013). Relationship of internet addiction with impulsivity and severity of psychopathology among Turkish university students. Psychiatry Res..

[29] Li W., Zhang W., Xiao L., Nie J. (2016). The association of internet addiction symptoms with impulsiveness, loneliness, novelty seeking and behavioral inhibition system among adults with attention-deficit/hyperactivity disorder (ADHD). Psychiatry Res..

[30] Wu X.-S., Zhang Z.-H., Zhao F., Wang W.-J., Li Y.-F., Bi L., Qian Z.-Z., Lu S.-S., Feng F., Hu C.-Y. (2016). Prevalence of internet addiction and its association with social support and other related factors among adolescents in China. J. Adolesc..

[31] Primack B.A., Escobar-Viera C.G. (2017). Social media as it interfaces with psychosocial development and mental illness in transitional age youth. Child Adolesc. Psychiatr. Clin. N. Am..

[32] Kim J., LaRose R., Peng W. (2009). Loneliness as the cause and the effect of problematic internet use: The relationship between internet use ans psychological weel-being. Cyberpsychol. Behav..

[33] Kraut R., Patterson M., Lundmark V., Kiesler S., Mukopadhyay T., Scherlis W. (1998). Internet paradox: A social technology that reduces social involvement and psychological well-being?. Am. Pdychologist.

[34] Wang K., Frison E., Eggermont S., Vandenbosch L. (2018). Active public Facebook use and adolescents ’felling of loneliness: Evidence for a curvilinear relationship. J. Adolesc..

[35] Li J., Zhan D., Zhou Y., Gao X. (2021). Loneliness and problematic mobile phone use among adolescents during the COVID-19 pandemic: The roles of escape motivation and self-control. Addict. Behav..

[36] Meade J. (2021). Mental health effects of the COVID-19 pandemic on children and adolescents. A review of the current research. Pediatric Clin..

[37] Loades M.E., Chatburn E., Higson-Swenney N., Reynolds S., Shafran R., Brigden A., Linney C., Niamh McManus M., Borwick C., Crawley E. (2020). Rapid systematic review: The impact of social isolation and loneliness on the mental health of children and adolescents in the context of COVID-19. J. Am. Acad. Child Adolesc. Psychiatry.

[38] Belfort E.L., Miller L. (2018). Relationship between adolescent suicidality, self-injury and media habits. Child Adolesc. Psychiatr. Clin. N. Am..

[39] Chen R., Liu J., Cao X., Duan S., Wen S., Zhang S., Xu J., Lin L., Xue Z., Lu J. (2020). The relationship between mobile phone use and suicide-related behaviors among adolescents: The mediating role of depression and interpersonal problems. J. Affect. Disord..

[40] Page M.J., McKenzie J.E., Bossuyt P.M., Boutron I., Hoffmann T.C., Mulrow C.D., Shamseer L., Tetslaff J.M., Akl E.A., Brennan S.E. (2021). The PRISMA 2020 statement: An updated guideline for reporting systematic reviews. Syst. Rev..

[41] Colder Carras M., Van Rooij A.J., Van de Mheen D., Musci R., Xue Q.-L., Mendelson T. (2017). Video gaming in a hyperconnected world: A cross-sectional study of heavy gaming, problematic gaming symptoms, and online socializing in adolescents. Comput. Hum. Behav..

[42] Shoshani A., Braverman S., Meirow G. (2021). Video games and close relations: Attachment and empathy as predictors of children’s and adolescent’s video game social play and socio-emotional functioning. Comput. Hum. Behav..

[43] Mozafar Saadati H., Mirzaei H., Okhovat B., Khodamoradi F. (2021). Association between internet addiction and loneliness across the world: A meta-analysis and systematic review. SSM Popul. Health.

